# Open-label, randomized, multi-center study comparing the sequence of high dose Aldesleukin (Proleukin^® ^(HD IL-2) and Ipilimumab Yervoy^® ^) in patients with metastatic melanoma (proclivity 02)

**DOI:** 10.1186/2051-1426-2-S3-P78

**Published:** 2014-11-06

**Authors:** Sapna Patel, Mohammed Milhem, Sigrun Hallmeyer, Gregory Daniels, Lee Cranmer, Bret Taback, Lawrence Flaherty, Sandra Aung, James Lowder, William Sharfman

**Affiliations:** 1 MD Anderson Cancer Center, Houston, TX, USA; 2 University of Iowa, Iowa City, IA, USA; 3 Oncology Specialists SC, Park Ridge, IL, USA; 4 Moores Cancer Center, La Jolla, CA, USA; 5 University of Arizona Cancer Center, Tucson, AZ, USA; 6 Columbia University, New York, NY, USA; 7 Karmanos Cancer Center, Detroit, MI, USA; 8 Prometheus Laboratories, San Deigo, CA, USA; 9 Johns Hopkins University, Baltimore, MD, USA

## Purpose

To investigate whether the sequence of HD IL-2 and a checkpoint inhibitor, Ipilimumab, will have additive or synergistic efficacy or toxicity when used in rapid sequence.

## Schema

**Figure 1:**
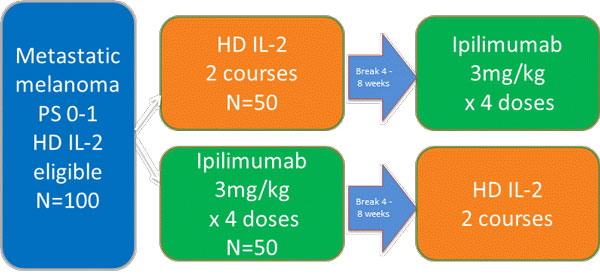
Open-label, randomized, multi-center study comparing the sequence of high dose Aldesleukin (Proleukin^® ^(HD IL-2) and Ipilimumab Yervoy^®^) in patients with metastatic melanoma (proclivity 02)

Adult patients with Stage IV or unresectable Stage III metastatic melanoma who are eligible to receive HD IL-2, treatment naïve or have received prior adjuvant therapy are randomized to a sequential administration of 4 doses of Ipilimumab or 4 cycles of HD IL-2 dosed according to their package inserts (figure 1). Fifty of the patients will start with one drug and 50 the other. The second drug will begin as soon as practically possible, without waiting for relapse. Entry criteria have recently been amended to include prior treatment with anti-PD-1 or anti-PDL-1. Twelve US sites are currently enrolling patients. An independent Data and Safety Monitoring Committee oversees the study. The primary endpoint is the proportional one year survival in the ITT population and a protocol defined population of patients who have received at least half of the planned doses of both study drugs. Clinical response and progression free survival will also be assessed. The primary endpoint is the proportional one year survival in the ITT population and a protocol defined population of patients who have received at least half of the planned doses of both study drugs. Clinical response and progression free survival will also be assessed.

## Current status

Twelve US sites are currently enrolling patients. To date 16 patients have been enrolled, 9 on the Ipilimumab first and 7 on the HD IL-2 first arms. No synergistic toxicity has been observed, but one death occurred in the HD IL-2 arm and one colectomy on the Ipilimumab arm, both prior to administration of the other drug.

